# Liquid Crystalline Siloxane-Containing Poly(ester imide)s with Low Dielectric Constant and Low Dielectric Loss at 10 GHz

**DOI:** 10.3390/polym18070782

**Published:** 2026-03-24

**Authors:** Qing Peng, Wenxiang Zhang, Qiwei Pan, Shumei Liu, Jianqing Zhao

**Affiliations:** 1 School of Materials Science and Engineering, South China University of Technology, Guangzhou 510640, China; 15574438924@163.com (Q.P.); wenxiang0825@126.com (W.Z.); psjqzhao@scut.edu.cn (J.Z.); 2 Guangdong Provincial Key Enterprise Laboratory of Novel Polyamide 6 Functional Fiber Materials Research and Application, Jiangmen 529100, China; 3 Key Laboratory of Polymer Processing Engineering, South China University of Technology, Ministry of Education, Guangzhou 510640, China

**Keywords:** poly(ester imide)s, siloxane, dielectric constant, dielectric loss, adhesion strength

## Abstract

The development of high-performance polymers exhibiting both low dielectric constant (D_k_) and low dielectric loss (D_f_) at high frequencies is highly desirable yet challenging for applications in microelectronics and wireless communication technologies. In this work, a series of siloxane-containing poly(ester imide)s (SiPEIs) are designed and synthesized via a two-step polymerization route, using 1,4-phenylene bis(1,3-dioxo-1,3-dihydroisobenzofuran-5-carboxylate) (TAHQ) as the dianhydride monomer, and 1,3-bis(3-aminopropyl)-1,1,3,3-tetramethyldisiloxane (DMS) together with 4,4′-diaminodiphenyl ether (ODA) as the diamine comonomers. Although the introduction of short siloxane segments lowers the glass transition temperature (*T*_g_) and the tensile strength of the resulting PEIs, they still remain at a relatively high level. Liquid crystalline phase behavior is observed at lower temperature for the siloxane-containing PEIs. Meanwhile, the hydrophobicity and the high-frequency dielectric performance is effectively improved with increasing siloxane content. Notably, SiPEI-20, prepared with 20 mol% DMS, displays an outstanding integrated performance. It exhibits a *T*_g_ of 200 °C, a D_k_ of 2.87 and a D_f_ of 0.00155 at 10 GHz, as well as an adhesive strength of 0.85 N·mm^−1^ on copper foil. Overall, this work provides a feasible strategy by incorporating siloxane into the PEI backbone, enabling the synergistic enhancement of high-frequency dielectric properties (simultaneous reduction in D_k_ and D_f_) and adhesion to copper foil.

## 1. Introduction

With the rapid development of 5G communication, the interlayer dielectric materials of flexible copper-clad laminate (FCCL) for 5G communication are required to further decrease the dielectric constant (D_k_) and dielectric loss (D_f_) in order to enhance the signal transmission speed and reduce the signal transmission loss. Polyimides (PIs) have been widely used in the microelectronics industry with their excellent performance and simple film-forming process [1]. However, the D_k_ of general PI (DuPont’s Kapton) is around 3.4, and D_f_ is around 0.014 at 10 GHz, which is far from meeting the requirements of 5G communication [2].

According to the famous Clausius–Mossotti equation, low molar polarization and large molar volume groups are needed to obtain a polymer with a low D_k_ [3]. Therefore, there are two major chemical structure strategies to reduce the D_k_ of PIs. One is the incorporation of fluorinated substitutes or alicyclic structure of low polarizability into PIs’ mainchain; the other is the introduction of large side groups in the PI structures, thus increasing the free volume and reducing the intermolecular interaction force [4]. These two strategies can effectively reduce the D_k_ of PIs, but they are not particularly effective in reducing the D_f_ to below 0.004 at 10 GHz [2,5].

D_f_ is extremely important for 5G or even higher frequency communication. Liquid crystalline polyester (LCP) exhibits a moderate D_k_ of 3.30, but a much lower D_f_ of 0.0028 at 10 GHz than that of PI [6]. Attributed to the prominent dielectric properties, LCP film is regarded as a promising substrate material for FCCL working in 5G communication [7]. In view of this, poly(ester imide)s (PEIs), combining the outstanding properties of both PIs and LCPs, have shown significant performance advantages and attracted great attention [8]. According to recent reports, the incorporation of phenyl-ester units into dianhydrides or diamines could effectively decrease the D_f_ of PIs. The rigid rod-like phenyl-ester or biphenyl structures are beneficial to induce the molecular chains to align closely along the in-plane direction of the film during imidization, which inhibits the motion of polarized groups and thus reduces the D_f_, as well as reducing the coefficient of thermal expansion (CTE) of the film [5,9,10]. Hasegawa et al. synthesize a series of ester-linked diamines and dianhydrides with different lengths and substituents to prepare PEIs. They show moderate D_k_ (3.2) and low D_f_ (0.003) at 10 GHz [9,10].

If the D_k_ and D_f_ of these PEIs can be further reduced, they will exhibit tremendous application prospects in millimeter-wave communication. A dielectric performance of D_k_ < 3.0 and D_f_ < 0.004 at GHz frequencies is assigned a relatively high rank (Rank 4) [10]. However, some of the factors governing these two properties are contradictory, making them difficult to improve simultaneously. For example, increasing the distance between the polymer chains can lead to a larger free volume, thereby reducing the D_k_, while the D_f_ tends to increase. Researchers have conducted extensive research work in this field. Currently, it is found that introducing trifluoromethyl groups into crosslinked PEI yields thin films with a D_k_ of 2.85 and a D_f_ of 0.00237, while the introduction of alicyclic structures reduces the D_k_ of PEI to 2.77 and lowers the D_f_ to as low as 0.0018 [11,12].

Copper-clad laminate is an important raw material for printed circuit boards (PCBs), acting as both the structural foundation and the medium for connecting various electronic components. Therefore, the adhesion strength between the polymer film and copper foil represents one of the key specifications when a polymer is used as an interlayer dielectric film in FCCL [13]. LCP films exhibit poor adhesion to ordinary copper foils [14]. Accordingly, the copper foils used for FCCL preparation require pre-treatment, which significantly increases process complexity and manufacturing costs. It can be inferred that the low polarity of LCP chains and the restricted mobility of rigid main chains are the primary reasons for this poor adhesion. Recently, few studies have focused on this critical issue. Therefore, the development of novel polymers with enhanced comprehensive properties via rational molecular design is of great scientific and practical significance, and remains an ongoing challenge in this field.

As is well known, the incorporation of siloxane units into the main chain of PIs can endow them with a variety of beneficial properties, such as good solubility, high hydrophobicity, excellent adhesion strength, and improved dielectric properties [15]. Due to the complexity of synthesizing siloxane-containing dianhydrides and the difficulty in their purification, siloxane-containing diamines are widely used in the preparation of PI films [16,17]. The introduction of flexible polydimethylsiloxane (PDMS) oligomeric segments into PI backbones via PDMS-containing diamines has been extensively investigated [18,19,20,21]. Shieh et al. synthesize a series of soluble PEIs from 2,2-bis(4-(4-aminophenoxy)phenyl)propane, amine-terminated PDMS (with an amine group equivalent weight of 450), and four types of ester-group-containing dianhydrides [18]. However, the presence of PDMS in PI main chains tends to induce microphase separation, resulting in two glass transition temperatures (*T*_g_s) [22,23,24].

**Scheme 1:**
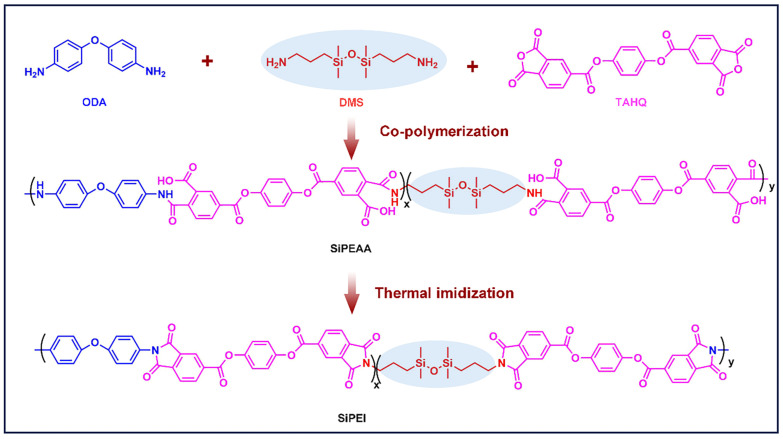
Synthetic route of the siloxane-containing poly(ester imide)s (SiPEIs).

In this work, an attempt is made to improve the high-frequency dielectric properties and adhesion strength to copper foil of PEIs. A series of siloxane-containing poly(ester imide)s (SiPEIs) are synthesized via a typical two-step thermal imidization process (Scheme 1), using an ester-group-containing dianhydride (1,4-phenylene bis(1,3-dioxo-1,3-dihydroisobenzofuran-5-carboxylate), TAHQ) and two diamine monomers, namely 4,4′-diaminodiphenyl ether (ODA) and 1,3-bis(3-aminopropyl)-1,1,3,3-tetramethyldisiloxane (DMS). The chemical structures, thermal and mechanical properties, phase behavior, dielectric and hydrophobic properties, and adhesion strength to copper foil of the resulting SiPEIs films are systematically investigated. To date, this is one of the few reported works capable of simultaneously decreasing D_k_ and D_f_ of the polymers at high frequency, as well as improving their adhesion with copper foil.

## 2. Materials and Methods

### 2.1. Materials

1,4-Phenylene bis(1,3-dioxo-1,3-dihydroisobenzofuran-5-carboxylate) (TAHQ), 4,4′-diaminodiphenyl ether (ODA), and 1,3-bis(3-aminopropyl)-1,1,3,3-tetramethyl disiloxane (DMS) were purchased from Aladdin Chemical Reagent Company, Ltd. (Shanghai, China). N,N-dimethylacetamide (DMAc) was purchased from Alfa Aesar Chemical Company, Ltd. (Shanghai, China). TAHQ was dried in a vacuum oven at 140 °C for 48 h prior to use. DMAc was dried over calcium hydride, and then distilled under reduced pressure to obtain anhydrous solvent.

### 2.2. Preparation of SiPEI Film

During the experiment, dianhydride and diamines were added at a molar ratio of 1.05:1, with the solid content maintained at 20 wt% and the reaction time set to 24 h. The experimental feed formulations are presented in Table 1. The polymers are denoted as SiPEI-n, where n is the molar fraction of DMS in diamines, varying at 0, 5%, 10%, 15%, and 20%.

Preparation of SiPEI-5 Films: 0.0245 g (0.1 mmol) of DMS, 0.380 g (1.9 mmol) of ODA, and 5.83 g of DMAC were weighed and added into a 25 mL three-necked flask. The solution was mechanically stirred in an ice-water bath. After the diamines were completely dissolved, 0.962 g (2.1 mmol) of TAHQ was added in one portion. The mixture was continuously stirred for 24 h under nitrogen. As the reaction proceeded, the viscosity of the solution gradually increased and its color darkened progressively, finally yielding a yellow and viscous siloxane-containing polyesteramic acid (PAA) solution. The solution was uniformly cast onto a horizontally placed glass plate, which was then placed in a vacuum oven at 25 °C for 4 h to remove residual bubbles from the PAA solution. Subsequently, thermal imidization was carried out in the vacuum oven following the programmed temperature protocol: 80 °C for 6 h, 120 °C for 2 h, 160 °C for 2 h, 200 °C for 2 h, and 250 °C for 1 h. After the glass plate was cooled to room temperature, it was immersed in hot water, and the SiPEI-5 film with a thickness of 40–80 μm was obtained.

### 2.3. Characterization

Fourier transform infrared spectra (FT-IR) were obtained from the transmission mode on a VERTEX70 spectrometer (Bruker, Germany). The inherent viscosities of SiPEIs were collected at 30 °C in DMAc at a concentration of 0.5 g dL^−1^ using an Ubbelohde viscometer. Thermogravimetric analysis (TGA) was performed under nitrogen atmosphere using a Netzsch TG 209F1 (Selb, Germany) at a heating rate of 20 °C·min^−1^. The glass transition temperature (*T*_g_) was determined by dynamic mechanical analysis (DMA-242C) at a heating rate of 5 °C·min^−1^ with a sinusoidal load frequency of 1 Hz under nitrogen atmosphere. The liquid crystalline morphology of SiPEIs was studied under a polarized optical microscope (STEMI 2000, ZEISS, Oberkochen, Germany) equipped with a hot stage. The mechanical property was assessed in accordance with ASTM D882-18 standard utilizing Instron 5967 model material testing equipment (USA), which was run at a strain rate of 5 mm·min^−1^ [25]. The linear coefficient of thermal expansion (CTE) was measured at a heating rate of 5 °C·min^−1^ as an average between 50 and 200 °C by thermomechanical analysis (TMA-402F3, Netzsch, Selb, Germany) with a stretch model at a preload force of 0.2 N. Dielectric properties were measured before and after moisture absorption at the frequency of 10 GHz using a cavity resonator perturbation technique using PNA-L network analyzer N5234B (Keysight, Santa Rosa, CA, USA). Water absorption was determined from the relation

W_A_ = (W − W_0_)/W_0_ × 100%,

where W_0_ is the weight of SiPEI film after vacuum-drying at 50 °C for 24 h, and W denotes the weight of SiPEI film which is immersed in water at 25 °C for 24 h and wiped with tissue paper. Adhesion property was tested by the T-peel strength test method with Instron 5967 model material testing equipment. The SiPEI film was dried in an oven at 120 °C for 2 h to remove surface moisture. The treated film was then laminated with 35 μm thick copper foil by hot pressing using a vacuum laminator at 300 °C and 20 MPa for 30 s under a vacuum of −0.1 MPa. The sample was cooled to below 80 °C under pressure and then removed. It was further heated in a vacuum oven at 200 °C for 1 h, and finally cut into strips.

## 3. Results and Discussion

### 3.1. Polymerization and Characterization of SiPEIs

As outlined in Scheme 1, SiPEI films are prepared via the conventional two-step polymerization procedure from ODA, DMS, and TAHQ with equivalent ratios of dianhydride to total diamines of 1.05:1. The detailed experimental formulae are listed in Table 1. Specifically, DMS accounted for 0 mol%, 5 mol%, 10 mol%, 15 mol%, and 20 mol% of the total diamine content, and the corresponding polymers were designated as SiPEI-0, SiPEI-5, SiPEI-10, SiPEI-15, and SiPEI-20.

**Figure 1:**
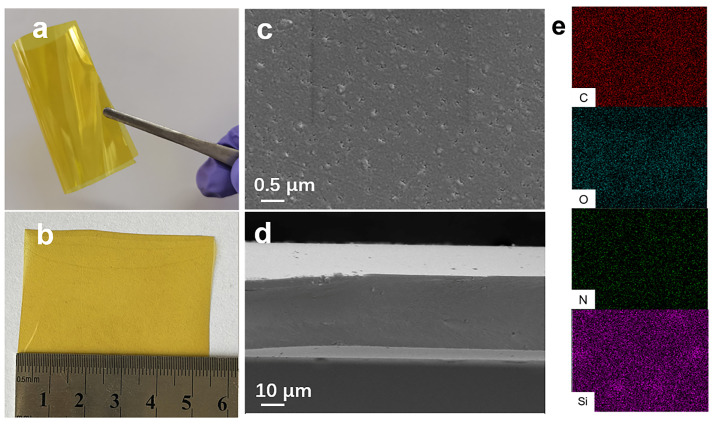
Photographs of the film of SiPEI-15 (**a**,**b**). The SEM images of the surface (**c**) and the cross-section (**d**) of the film. EDS elemental mapping of the surface of the film (**e**).

The copolymerization between dianhydride and two diamines is carried out in an ice-water bath because the reaction to form siloxane-containing polyesteramic acids (SiPEAAs) is reversible and exothermic. Lowering the temperature facilitates the molecular weight growth. During polymerization, all systems with a solid content of 20 wt% exhibit the “rod-climbing” gel phenomenon, indicating that high-molecular-weight polymers are obtained. Then tough and flexible SiPEI films are obtained by casting the SiPEAA solution on a glass plate followed by thermal imidization. The SiPEI-15 film exhibits light yellow color and good flexibility, as shown in Figure 1a,b. Both the surface and cross-section of the film of SiPEI-15 are observed with SEM. Images are shown in Figure 1c,d. The film is relatively smooth, without any obvious phase separation. The corresponding elemental mapping of C, O, N, and Si on the surface of the film via EDS (Figure 1e) also confirms the uniformity of the as-prepared SiPEI-15 film.

SiPEIs are almost insoluble in all organic solvents; thus, their molecular weights are indirectly characterized by measuring the viscosities of their soluble intermediates, SiPEAAs. The specific data are presented in Table 1. The inherent viscosities (*η*_inh_) range from 1.96 to 1.65 dL/g, indicating the successful preparation of high-molecular-weight polymers. Although the values show a slight downward trend with increasing content of the flexible siloxane segments in the system, this phenomenon is speculated not to arise from the reduction in polymer molecular weight. The primary cause may lie in the high flexibility of Si-O and Si-C bonds, which reduces the rigidity of the molecular chains, improves chain mobility, and decreases interchain friction, consequently resulting in lower viscosity. A similar phenomenon is also reported by Lee et al., who prepare a series of siloxane-containing copolyimides based on 3,3′,4,4′-benzophenone tetracarboxylic dianhydride, ODA, and DMS. As the molar ratio of DMS to ODA varied from 0:10 to 3:8, the *η*_inh_ decreased from 1.25 dL·g^−1^ to 0.80 dL·g^−1^ [26].

**Figure 2:**
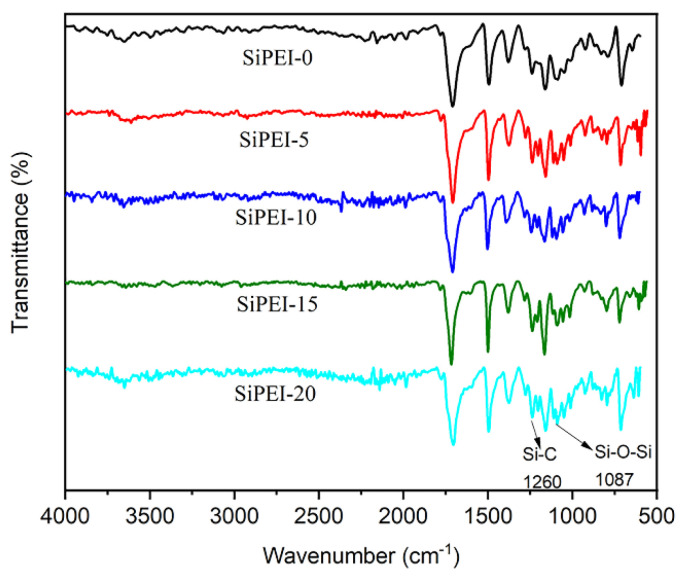
FT-IR spectra of the SiPEIs.

The chemical structure and imidization degree of the samples are confirmed by FT-IR (Figure 2). The characteristic absorption peaks at 1780 cm^−1^, 1715 cm^−1^, 1370 cm^−1^ and 730 cm^−1^ are assigned to the symmetrical stretching of C=O bonds, the asymmetrical stretching of C=O bonds, C-N stretching vibration, and imide bending vibration, respectively. Moreover, the peaks at 1640 cm^−1^ (the C=O stretching of amide) and 3345–3356 cm^−1^ caused by N-H stretching disappear. The lack of the amide-related bands and the appearance of the corresponding imide bands indicate the complete imidization of all samples. In addition, the absorption bands at 1087 cm^−1^, 1023 cm^−1^ (asymmetric stretching of Si-O-Si), and 1260 cm^−1^ certify the successful introduction of siloxane units into the main chain of PEIs.

### 3.2. Thermal and Mechanical Characterization of the SiPEIs

**Figure 3:**
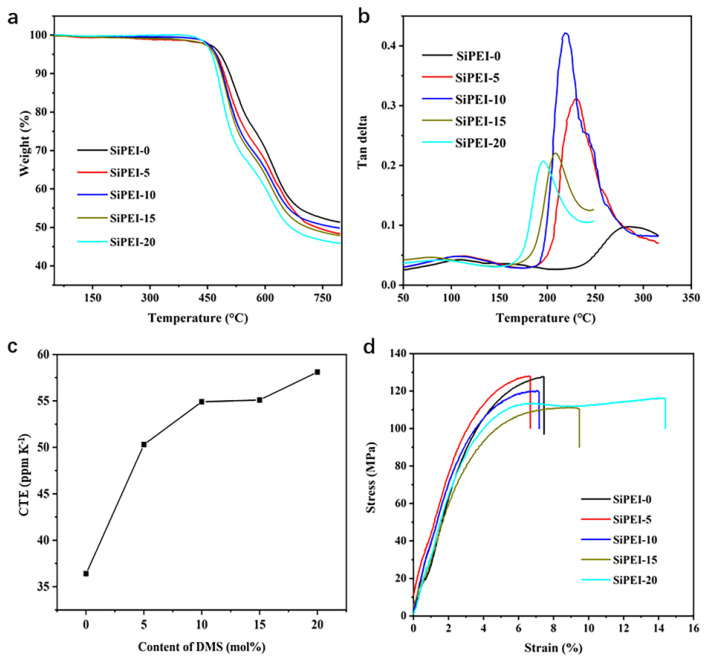
(**a**) TGA curves of the SiPEIs. (**b**) Tan delta vs. temperature curves of the SiPEIs. (**c**) CTE values of the SiPEIs with different DMS contents. (**d**) Stress–strain curves of the SiPEIs.

The thermal stability of the SiPEI films is characterized by thermogravimetric analysis (TGA), and the curves are presented in Figure 3a. The thermal stability of the samples is evaluated by the temperatures of 5% and 10% weight loss (*T*_d_^5%^ and *T*_d_^10%^), as well as the residual mass (R_w_) at 800 °C, with the corresponding data summarized in Table 2. With increasing temperature, the main chains of the SiPEIs undergo thermal decomposition accompanied by the cleavage of the aliphatic segments in the main chain, leading to a rapid mass loss. The incorporation of siloxane segments results in a certain decrease in both the *T*_d_^5%^ and *T*_d_^10%^ values. Compared with SiPEI-0, when the siloxane content reaches 20 mol%, *T*_d_^5%^ of SiPEI-20 decreases from 484 °C to 463 °C, and *T*_d_^10%^ declines from 508 °C to 480 °C. Upon further heating to 800 °C, the R_w_ of SiPEI-0 is 51.4%. All other samples exhibit decreasing values lower than 50%.

The glass transition temperatures (*T*_g_) of SiPEIs are determined by dynamic thermomechanical analysis (DMA, Figure 3b). The peak of the loss factor (tanδ) curve corresponds to *T*_g_ (Table 2). As can be seen from the results, SiPEI-0 exhibits a relatively high *T*_g_ of 287 °C. Aromatic PIs possess a large delocalized conjugated structure, strong molecular chain polarity, and a rigid, regular backbone. These features lead to strong intermolecular interactions and restricted mobility of the rigid main chains, endowing the polymer with extremely high *T*_g_ values. However, the incorporation of siloxane gradually disrupts the rigid delocalized structure. Siloxane segments consist of flexible single-bonded chains; in particular, the Si-O-Si linkage exhibits a low rotational energy barrier and high rotational freedom. This disrupts the tight packing of the molecular chains and weakens intermolecular forces, resulting in a progressive decrease in the *T*_g_ of the samples. With the introduction of 5% DMS, *T*_g_ of SiPEI-5 decreases to 232 °C. The *T*_g_ of SiPEI-20 further decreases to 200 °C.

SiPEIs are characterized by the coefficient of linear thermal expansion (CTE), which is measured using a thermomechanical analyzer (TMA) over the temperature range of 50–200 °C. The results are summarized in Table 2, with the corresponding trend illustrated in Figure 3c. These SiPEIs exhibit moderate CTE values ranging from 36.4 to 58.1 ppm·K^−1^. As the molar ratio of DMS in the diamine monomers increases from 0 to 20 mol%, the CTE values increase by 60%. This demonstrates that the introduction of siloxane units enhances molecular chain flexibility and impairs dimensional stability, typically resulting in higher CTE values.

The SiPEI films prepared via thermal imidization are light yellow, flexible, and tough. Their mechanical properties are measured, with the results listed in Table 2, and the stress–strain curves are depicted in Figure 3d. For all samples, the mechanical properties generally follow a consistent trend: as siloxane content increases, the tensile strength of the SiPEIs gradually decreases, while the elongation at break increases correspondingly. However, Young’s modulus increases first, and then decreases. Specifically, the modulus and tensile strength of SiPEI-20 are 3.4 GPa and 116.7 MPa, respectively, which are lower than those of SiPEI-0 (4.7 GPa and 128.1 MPa, respectively). The elongation at break of the SiPEI-15 and SiPEI-20 films increased to 9.5% and 14.4%, representing increments of 28.7% and 94.6%, respectively, compared with SiPEI-0. The variations in mechanical properties primarily originate from the incorporation of highly flexible DMS segments between the rigid PEI structural units. This incorporation increases the free volume of the polymer chains, weakens intermolecular forces, and thus reduces the mechanical strength. Meanwhile, the flexible siloxane segments facilitate the movement and deformation of molecular chains, resulting in an improvement in the elongation at break of the samples.

Although *T*_d_^5%^, *T*_g_, tensile strength, and Young’s modulus of the SiPEIs are reduced to a certain extent due to the introduction of flexible siloxane units, the overall thermal and mechanical properties of these SiPEIs remain at a relatively high level. These films exhibit excellent toughness and strength, making them potential candidates for applications in flexible copper-clad laminates (FCCLs) and flexible displays.

### 3.3. Phase Behaviors of SiPEIs

**Figure 4:**
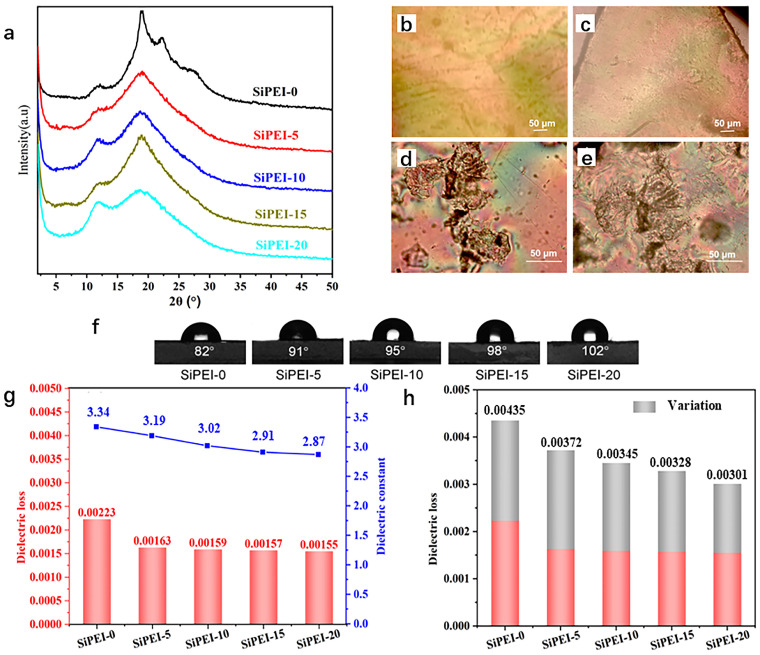
(**a**) XRD patterns of SiPEIs at room temperature. POM images of SiPEI-5 at 315 °C (**b**), SiPEI-10 at 300 °C (**c**), SiPEI-15 at 290 °C (**d**), and SiPEI-20 at 250 °C (**e**). Water contact angles of SiPEI films (**f**). Dielectric properties of SiPEIs film at 10 GHz (**g**), and change in dielectric loss tangent of the films at 10 GHz after moisture absorption (**h**).

To investigate the effect of siloxane units on the crystalline phase, the aggregation structure of SiPEIs is studied using X-ray powder diffraction (XRD). Figure 4a presents the XRD patterns of the samples at room temperature. SiPEI-0 exhibits four sharp diffraction peaks in the 2θ range of 10–30°, indicating that the introduction of aromatic ester groups significantly enhances the crystallinity of PIs. However, with the incorporation of siloxane units, the crystallinity of the SiPEIs decreases rapidly. The diffraction peak at around 2θ = 11.2° remains, and one broad diffraction peak appears in the 2θ range of 15–30° in their XRD patterns, with its position shifting slightly to the left with increasing siloxane content. The specific diffraction data are summarized in Table 3. Calculations based on Bragg’s equation show that the interchain distances (*d*) of the polymers increase slightly, with values of 4.68 Å and 4.75 Å for SiPEI-0 and SiPEI-20, respectively. This result indicates that the interchain distance of the SiPEIs gradually enhances with increasing siloxane content, leading to an expansion of the free volume. Therefore, the introduction of siloxane units is favorable for reducing the dielectric constant of the samples.

The densities of the SiPEIs are determined using an electronic densimeter, with the detailed values presented in Table 3. Compared with SiPEI-0 (density = 1.25 g/cm^3^), the densities of SiPEI-5, SiPEI-10, SiPEI-15, and SiPEI-20 decrease to 1.24 g/cm^3^, 1.13 g/cm^3^, 1.13 g/cm^3^, and 1.12 g/cm^3^, respectively, with a reduction range of less than 10%. This suggests that the incorporation of siloxane segments into the main chain increases the intermolecular distance between SiPEI chains, thereby resulting in a lower packing density of the samples. In general, a decrease in the density of PIs is accompanied by an increase in the air content within the matrix, which tends to lead to a reduction in the dielectric constant.

The para-phenyl ester units in the PEI main chain provide sufficient structural rigidity and linearity, along with a relatively high degree of in-plane chain orientation [27,28]. Therefore, TAHQ as a typical tetracarboxylic dianhydride linked by para-phenyl ester groups is commonly used for the synthesis of various PEIs. However, the liquid crystalline phases of whole aromatic PEIs are rarely detected, mainly because the molecular chains possess high rigidity and their melting points are higher than their thermal decomposition temperatures, making it impossible to study their phase behavior. After introducing short siloxane units into the main chain, the chain rigidity decreases, enabling the formation of liquid crystalline phases at lower temperatures. As shown in the polarized optical microscopy (POM) images in Figure 4b–e, SiPEI-5, SiPEI-10, SiPEI-15, and SiPEI-20 exhibit obvious birefringence at 315 °C, 300 °C, 290 °C, and 250 °C, respectively, indicating the formation of a liquid crystalline state. It can be concluded that the formation of the liquid crystalline phase requires a certain degree of mobility of the rigid chains. The higher the siloxane content, the lower the temperature at which the liquid crystalline phase is observed. Gu et al. also report a series of thermotropic liquid crystalline PIs by adjusting the ratio of rigid mesomorphic units (phthalimide groups) and flexible groups (ether bonds) [29]. The SiPEI-20 shows a smectic phase at 300 °C (Figure S1).

### 3.4. Hydrophobic and High-Frequency Dielectric Properties of SiPEIs

Hydrophobicity is one of the key properties of high-frequency communication substrates. PI films with poor hydrophobicity tend to delaminate and warp from copper foil and other substrates in hot and humid environments, which seriously degrades the quality and reliability of electronic devices. The hydrophobic behavior of SiPEIs is investigated via water absorption and water contact angle measurements. Water absorption data in Table 3 indicates that increasing the siloxane content in SiPEIs leads to a downward trend in the values. The water absorption of SiPEI-0 is 0.70%, which decreases to 0.42% for SiPEI-20.

The water contact angle of the films shows an increasing trend with increasing siloxane content (Figure 4f and Table 3). The values for SiPEI-0, SiPEI-5, SiPEI-10, and SiPEI-15 are 82°, 91°, 95°, and 98°, respectively. Notably, the surface water contact angle of SiPEI-20 reaches as high as 102° at a siloxane content of 20 mol%. Overall, the variation trend of the contact angle is basically consistent with that of water absorption, and the as-prepared SiPEI films exhibit excellent hydrophobicity, which is attributed to the low surface energy and low-polarity structures of siloxane units in the SiPEI backbones.

Polymers with low D_k_ and low D_f_ at high frequencies are particularly critical for substrate materials in high-speed communication, as they are highly beneficial for increasing the signal transmission speed and reducing the signal transmission loss of microelectronic devices. The D_k_ and D_f_ of SiPEIs at 10 GHz are investigated, with the results presented in Figure 4g. The D_k_ values of the SiPEIs decrease with increasing siloxane content, with values of 3.34, 3.19, 3.02, and 2.91 for SiPEI-0, SiPEI-5, SiPEI-10, and SiPEI-15, respectively. For SiPEI-20 with a higher siloxane content, the D_k_ is 2.87, representing a 14% reduction compared with SiPEI-0.

According to the Clausius–Mosotti equation, the factors influencing the D_k_ of polymers mainly include the polarity of molecular chains, interchain distance, and packing regularity of molecular chains. Conventional aromatic PIs exhibit highly rigid structures, good main-chain axial symmetry, strong intermolecular forces between chain segments, tight molecular chain packing, and high polarity; these characteristics result in a small molar volume and high molar polarizability, leading to a D_k_ of approximately 3.4. However, the introduction of nonpolar siloxane units effectively reduces the molar polarizability. On the other hand, the rigid structural units derived from ODA and TAHQ monomers are randomly distributed with the siloxane segments, which increases the interchain distance and free volume of the polymers. The synergistic effect of these factors contributes to the reduction in D_k_ values of the SiPEIs.

At high frequencies (>1 GHz), the high D_f_ of conventional PIs (>0.01) is primarily attributed to their high water absorption and strong dipolar polarization induced by imide rings. Recent studies have reported that PEIs bearing rod-like phenyl-ester units exhibit a low D_f_ of approximately 0.003 at 10 GHz, which is ascribed to their reduced imide content and water absorption, together with the retention of a densely packed rigid polymer chain structure [9,10]. As illustrated in Figure 4g, with an increase in siloxane content in SiPEIs, the D_f_ values of the polymers gradually decrease, with respective values of 0.00223, 0.00163, 0.00159, 0.00157, and 0.00155 for SiPEI-0, SiPEI-5, SiPEI-10, SiPEI-15, and SiPEI-20, respectively.

**Figure 5:**
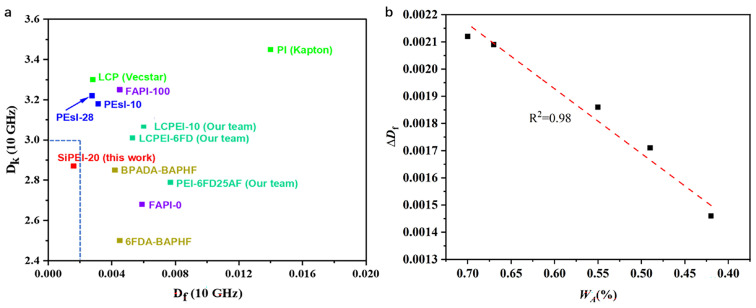
D_k_ and D_f_ of modified PIs, LCP and SiPEI-20 (**a**). PI, BPADA-BAPHF and 6FDA-BAPHF are from ref. [2]. FAPI-100 and FAPI-0 are from ref. [5]. LCP is from ref. [6]. PEsI-10 is from ref. [9]. PEsI-28 is from ref. [10]. LCPEI-10 is from ref. [30]. LCPEI-6FD and PEI-6FD25AF are from ref. [31]. The correlation curve between the D_f_ variation of SiPEIs and the water absorption values (**b**).

It is worth noting that both D_k_ and D_f_ of the SiPEIs decrease gradually with the increasing siloxane content in the polymer backbones. When the feeding ratio of DMS in diamines is up to 20 mol%, the D_k_ and D_f_ are decreased to 2.87 and 0.00155 at 10 GHz, respectively. In most of the cases, introduction of soft or bulky segments into the PEIs leads to declining D_k_, but increasing D_f_, owing to their disruption of the dense stacking of the polymer chains [12,30]. Simultaneous reduction of both D_k_ and D_f_ is challenging. Figure 5a shows the dielectric properties of the modified PI and LCP films at 10 GHz. As can be seen from the figure, FAPI-0 prepared by Yang et al. [5] from 4,4′-(hexafluoroisopropylidene)diphthalic anhydride (6FDA), 2,2′-bis(trifluoromethyl)benzidine (TFDB), and 3,3′,4,4′-biphenyltetracarboxylic dianhydride (BPDA) exhibits a moderate D_f_ (0.0059) and low D_k_ (2.68). PI prepared by Kuo et al. [2] from bisphenol A-type diether dianhydride (BPADA) and fluorinated diamine BAPHF exhibits a D_k_ of 2.85 and D_f_ of 0.0042, while the PI based on 6FDA and BAPHF shows a D_k_ of 2.50 and D_f_ of 0.0045. Commercially available Kapton film [2] has a D_k_ of 3.45 and D_f_ of 0.014, while polyester LCP (Vecstar) film [6] has a D_k_ of 3.30 and D_f_ of 0.0028. PEIs prepared by Hasegawa et al. [10] from longitudinally extended ester-linked tetracarboxylic dianhydride (TA-44BPHB), p-phenylenediamine (p-PDA), and ODA show a moderate D_k_ (3.22) and low D_f_ (2.76 × 10^−3^). LCPEI-10, a liquid crystalline polyester imide prepared via solution polymerization by our group from 4-hydroxybenzoic acid (HBA), isophthalic acid (IPA), 4,4′-dihydroxybiphenyl (BP), and 4,4′-dihydroxydiphenyl ether (ODP), has a D_k_ of 3.07 and D_f_ of 0.0060 [30]. Fluorinated LCPEI-6FD, copolymerized from fluorine-containing imide dicarboxylic acid (IA), IPA, HBA, and bisphenol monomer, possesses moderate D_f_ (0.0053) and D_k_ (3.01) [31]. For comparison, the SiPEI-20 film prepared in this work exhibits a D_f_ below 0.002 and a D_k_ below 3.0, demonstrating superior dielectric properties at high frequencies compared with other modified PI and LCP films.

In this work, DMS is an aliphatic amine with four methyl groups symmetrically arranged on the sides of the siloxane group, which leads to increased molar volume and decreased molar polarization of the PI chains [32,33]. Although the distance of the SiPEI main chains enlarges, the short siloxane units improve the hydrophobicity of the polymers and give the rigid rod-like chains some mobility to form liquid crystalline phases. Therefore, both D_k_ and D_f_ of the SiPEIs decrease.

Moisture can impair the dielectric properties of materials. In actual service environments, humidity and temperature exert a significant influence on the properties of PI films, mainly because they absorb water in humid environments, thereby increasing their dielectric constant and dielectric loss. To better illustrate the effect of moisture absorption on the high-frequency dielectric properties of SiPEIs, each film is placed in a chamber at 25 °C and 50% RH for 24 h, and then its D_k_ and D_f_ at 10 GHz are measured. The detailed data are listed in Figure 4h and Table S1. The variation in D_k_ for SiPEI films is less than 3%. However, the effect of moisture on D_f_ is significantly greater, and D_f_ increases to 0.00435, 0.00372, 0.00345, 0.00328, and 0.00301 for SiPEI-0, SiPEI-5, SiPEI-10, SiPEI-15, and SiPEI-20, respectively. Ionic conduction, dipole relaxation, and interfacial polarization induced by moisture all contribute to the increase in D_f_ [34,35]. With enhancing siloxane content, the increment of D_f_ for SiPEIs decreases. The possible reason is that the introduction of siloxane moieties effectively reduces the water absorption of the samples, thus weakening the influence of humid environments on the dielectric properties of the films.

The correlation curve between the D_f_ variation of SiPEIs and the water absorption of the polymers is fitted (Figure 5b). The D_f_ variation decreases linearly with the reduction in water absorption of the films, with a correlation coefficient of 0.98. Therefore, ensuring a low water absorption of the material is crucial for the stability of its dielectric properties in practical applications.

### 3.5. Adhesion Property

Adhesion strength of polymer dielectric films with copper foil substrates is an important performance indicator in FCCL applications [13]. The testing samples are prepared by hot pressing SiPEI film with the copper foil; the peel strengths are measured and summarized in Table 3. Obviously, the siloxane-containing PEIs exhibit better adhesive strength than SiPEI-0 (0.60 N·mm^−1^). The adhesive strengths of SiPEI-5, SiPEI-10, and SiPEI-15 to copper foil are 0.72 N·mm^−1^, 0.77 N·mm^−1^, and 0.80 N·mm^−1^, respectively, all of which exceed the industrial standard of 0.70 N·mm^−1^. As the feeding ratio of siloxane-diamine increases to 20%, the adhesive strength increases by 42% to 0.85 N·mm^−1^.

On the one hand, the siloxane units with low surface energy can readily diffuse to the surface of the copper foil. EDS results (Figure 1e) reveal that Si element is uniformly distributed on the surface, which is beneficial to improve the adhesive strength. Li et al. synthesize a series of fluorinated poly(imide siloxane) based on 4,4′-(hexafluoroisopropylidene)diphthalic anhydride, 2,2′-bis(trifluoromethyl)-[1,1′-biphenyl]-4,4′-diamine and DMS. The introduction of siloxane segments significantly enhances the adhesive properties of the copolymers [36]. On the other hand, the introduction of flexible siloxane segments enhances the mobility of PEI chains under identical lamination conditions. At elevated temperature and pressure, the polymer chains can be more readily “squeezed into” the gaps of the copper foil, thereby improving the adhesion between the sample film and the copper plate [13]. In contrast, SiPEI-0 exhibits rigid molecular chains and a densely packed structure. Even under high temperature and pressure, it remains difficult to form effective physical interlocking with the copper foil.

## 4. Conclusions

A series of siloxane-containing poly(ester imide)s (SiPEIs) is successfully synthesized via a facile two-step copolymerization method, using 1,4-phenylene bis(1,3-dioxo-1,3-dihydroisobenzofuran-5-carboxylate) (TAHQ) as the ester-containing dianhydride monomer, bis(aminopropyl)tetramethyldisiloxane (DMS) as the organosiloxane unit, and 4,4′-oxydianiline (ODA) as the comonomer. At 10 GHz, both D_k_ and D_f_ of the SiPEI tend to decrease with increasing siloxane content. The D_k_ and D_f_ values of the non-siloxane sample SiPEI-0 are 3.34 and 0.00223, which decrease to 2.87 and 0.00155 for SiPEI-20, corresponding to a 14% reduction in D_k_ and a 30% decline for D_f_. It is observed that the introduction of the soft nonpolar siloxane units reduces the polarity of the polymer chains and increases their free volume, leading to decreasing D_k_, while the presence of siloxane greatly enhances the hydrophobicity of the polymers, and induces a liquid crystalline phase, which also results in a reduction in the D_f_ value. In addition, the introduction of short siloxane segments enhances the adhesion of SiPEI film to copper foil, leading to increased peel strength. The peel strength of SiPEI-20 is 0.85 N·mm^−1^, which is 42% higher than that of SiPEI-0 (0.60 N·mm^−1^).

All SiPEIs show high thermal stability, with the *T*_d_^5%^ above 463 °C. Although *T*_g_ and tensile strength decrease with increasing siloxane content, they keep at a relatively high level. The *T*_g_s are above 200 °C. The tensile modulus and the tensile strength are in the ranges of 3.4–5.5 GPa and 112.8–128.1 MPa, respectively. The elongation at break and CTE of the samples increase with the rise in silicon content, reaching 6.7–14.4% and 36.4–58.1 ppm·K^−1^, respectively. In summary, the introduction of siloxane short chains not only simultaneously reduces the D_k_ and D_f_ values of PEI, but also enhances its adhesion to copper foil. Meanwhile, the excellent thermal and mechanical properties of PEI are maintained to a certain degree, indicating its promising application potential in the field of flexible copper-clad laminates.

## Figures and Tables

**Table 1:** Recipes (mol ratio) of the SiPEIs and the inherent viscosities of the SiPEAAs.

Samples	TAHQ	ODA	DMS	*η*_inh_ ^a^ (dL·g^−1^)
SiPEI-0	10.5	10.0	0	1.96
SiPEI-5	10.5	9.5	0.5	1.89
SiPEI-10	10.5	9.0	1.0	1.83
SiPEI-15	10.5	8.5	1.5	1.79
SiPEI-20	10.5	8.0	2.0	1.65

^a^ Inherent viscosity measured in DMAc at a concentration of 0.5 g dL^−1^ at 30 ± 0.1 °C.

**Table 2:** Thermal and mechanical properties of the SiPEIs.

Sample	*T*_g_ ^a^ (°C)	*T*_d_^5%^ (°C)	*T*_d_^10%^ (°C)	R_w_	CTE (ppm·K^−1^)	Young’s Modulus (GPa)	Tensile Strength (MPa)	Elongation at Break (%)
SiPEI-0	287	484	507	51.4	36.4	4.7	128.1	7.4
SiPEI-5	232	473	493	48.3	50.3	5.5	124.2	6.7
SiPEI-10	222	472	489	49.8	54.9	5.3	120.8	7.2
SiPEI-15	209	468	486	47.9	55.1	3.6	112.8	9.5
SiPEI-20	200	463	480	45.9	58.1	3.4	116.7	14.4

^a^ *T*_g_ was measured by DMA.

**Table 3:** XRD data, hydrophobicity, and adhesion to copper foils of the SiPEIs.

Sample	2θ (°)	*d* (Å)	Density (g/cm^3^)	Water Absorption (%)	Contact Angle (°)	Peel Strength (N·mm^−1^)
SiPEI-0	19.0	4.68	1.25	0.70	82	0.60
SiPEI-5	18.9	4.69	1.24	0.67	91	0.72
SiPEI-10	18.8	4.73	1.13	0.55	95	0.77
SiPEI-15	18.7	4.74	1.13	0.49	98	0.80
SiPEI-20	18.6	4.75	1.12	0.42	102	0.85

## Data Availability

The original contributions presented in this study are included in the article/Supplementary Materials. Further inquiries can be directed to the corresponding authors.

## Supplementary Materials

The following supporting information can be downloaded at https://www.mdpi.com/article/10.3390/polym18070782/s1: Figure S1. (a) 2D-WAXD pattern of SiPEI-20 at 300 °C. (b) Intensity profile along the meridian direction of the 2D-WAXD pattern of SiPEI-20. Table S1. Dielectric properties at 10 GHz of the SiPEIs before and after moisture absorption.
