# Supplementary material for: Liquid Crystalline Siloxane-Containing Poly(ester imide)s with Low Dielectric Constant and Low Dielectric Loss at 10 GHz
Source: Polymers (Basel). 2026 Mar 24;18(7):782. doi: 10.3390/polym18070782 (PMC13074429; doi:10.3390/polym18070782)
Supplement: Supplementary file 1 [file polymers-18-00782-s001.zip › polymers-4196069-supplementary.pdf]

Liquid Crystalline Siloxane-Containing Poly(ester imide)s with Low Dielectric Constant and Low Dielectric Loss at 10 GHz

Qing Peng [1,†], Wenxiang Zhang [1,†], Qiwei Pan [1,2,*], Shumei Liu [1,3,*] and Jianqing Zhao [1,3]

[1] School of Materials Science and Engineering, South China University of Technology, Guangzhou 510640, China
[2] Guangdong Provincial Key Enterprise Laboratory of Novel Polyamide 6 Functional Fiber Materials Research and Application, Jiangmen 529100, China
[3] Key Laboratory of Polymer Processing Engineering, South China University of Technology, Ministry of Education, Guangzhou 510640, China
*Correspondence: panqw@scut.edu.cn (Q.P.); liusm@scut.edu.cn (S.L.)
[†] These authors contributed equally to this work.

To further investigate the phase structure of the SiPEIs, the two dimensional wide-angle X-ray diffraction (2D-WAXD) pattern of SiPEI-20 at 300 °C is obtained and shown in Figure S1a, where the unaligned film is heated directly, and the X-ray incident beam is perpendicular to the film. Three ring patterns are observed. The intensity profile along the meridian direction of the 2D-WAXD pattern of SiPEI-20 is presented in Figure S1b. In the low 2θ region, diffraction rings are observed at 2θ = 3.3° (d_1 = 2.67 nm) and 2θ = 6.6° (d_2 = 1.34 nm), while a distinct diffraction peak appears in the high 2θ region at 2θ = 19.0° (*d*-spacing = 0.47 nm). Given that d_1:d_2 = 2:1, the WAXD pattern confirms that SiPEI-20 exhibits a smectic liquid crystalline phase.

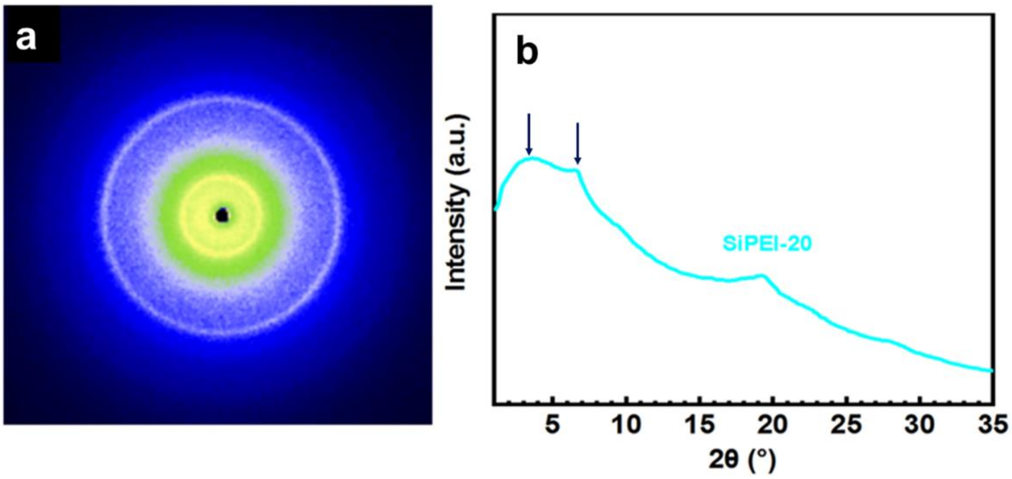

Figure S1. (a) 2D-WAXD pattren of SiPEI-20 at 300 °C. (b) Intensity profile along the meridian direction of the 2D-WAXD pattern of SiPEI-20.

The dielectric properties at 10 GHz of the SiPEIs before and after moisture absorption are summarized in Table S1. The dielectric loss (D_f) increases significantly after moisture uptake, and its variation gradually decreases with increasing siloxane content. By contrast, the dielectric constant (D_k) exhibits only a slight increase, with a relative change of less than 3.0%. The rate of change for D_f ranges from 87.7% to 128.2%, and it decreases progressively from SiPEI-5 to SiPEI-20.

Table S1. Dielectric properties at 10 GHz of the SiPEIs before and after moisture absorption.

Samples	D_k	D_f	D_k’	D_f’	ΔD_k	ΔD_f	[a]ΔD_k (%)	[b]ΔD_f (%)
	Dried at 105 °C for 2 h		after 25 °C×50% RH for 24 h					
SiPEI-0	3.34	0.00223	3.39	0.00435	0.05	0.00212	1.5	94.2
SiPEI-5	3.19	0.00163	3.25	0.00372	0.06	0.00209	1.9	128.2
SiPEI-10	3.02	0.00159	3.11	0.00345	0.09	0.00186	3.0	120.1
SiPEI-15	2.91	0.00157	2.93	0.00328	0.02	0.00171	0.7	111.5
SiPEI-20	2.87	0.00155	2.90	0.00301	0.03	0.00146	1.0	87.7

[a] $\Delta D_k = (D_k' - D_k)/D_k \times 100\%$; [b] $\Delta D_f = (D_f' - D_f)/D_f \times 100\%$
